# Combining motivational and volitional approaches to reducing excessive alcohol consumption in pre-drinkers: a theory-based intervention protocol

**DOI:** 10.1186/s12889-015-2648-7

**Published:** 2016-01-16

**Authors:** Kim M. Caudwell, Barbara A. Mullan, Martin S. Hagger

**Affiliations:** Health Psychology & Behavioural Medicine Research Group, School of Psychology and Speech Pathology, Curtin University, Perth, Australia

**Keywords:** Pre-drinking, Alcohol consumption, Theory-based intervention, Autonomy support, Implementation intention, Theoretical integration

## Abstract

**Background:**

Pre-drinking refers to the consumption of alcohol at home or a private residence prior to attending a subsequent social event. We present the study protocol of an online theory-based intervention to reduce pre-drinking and related harm in pre-drinking undergraduates, using behavior change techniques targeting the motivational and volitional phases of behaviour.

**Design:**

A fully randomized 2 (autonomy support: present vs. absent) x 2 (implementation intention: present vs. absent) between-participants design will be used to ascertain the effectiveness of the intervention in reducing pre-drinking alcohol consumption and alcohol-related harm. Participants will complete a range of theory-based measures prior to being allocated to one of the four experimental conditions. Four weeks later, participants will complete a follow-up questionnaire comprised of theoretical and behavioral measures.

**Analyses:**

The main and interactive effects of the intervention components in reducing our primary dependent variables, namely, pre-drinking alcohol consumption and alcohol-related harm at four-week follow-up will be tested. Baseline alcohol consumption and demographic information will be included in the analysis as covariates.

**Discussion:**

This online intervention is the first to be developed to reduce pre-drinking alcohol consumption, a behaviour linked to increased risk of alcohol-related harm. The intervention targets motivational and volitional components of the behaviour change process and is therefore likely to lead to greater reductions in pre-drinking alcohol consumption and experience of alcohol-related harm compared to either approach in isolation. If successful, the intervention can be implemented across various contexts and in populations where pre-drinking is prevalent.

**Trial registration:**

ACTRN12614001102662. Registered 16 October 2014.

## Background

Excessive alcohol consumption is associated with increased risk of acute (e.g., accidental injury) and chronic (e.g., cardiovascular disease, cancers, diabetes, liver disease, alcohol dependence, and a range of mental health conditions) harms [1]. In Australia, national costs of excessive alcohol consumption is estimated at 15 billion dollars annually, attributed to decreased workplace productivity, strain on the healthcare system, road or vehicular accidents, crime and associated costs, illness, and death [2]. Excessive alcohol consumption is especially apparent in university populations, with a third of students drinking to hazardous levels [3, 4] and appearing to outdrink their non-student peers on drinking occasions [5, 6]. Research shows that excessive alcohol consumption significantly impairs students’ health and academic performance, and increases risk-taking behaviors such as unplanned sexual activity [7].

Recent research has focussed on *pre-drinking*, the practice of consuming alcohol prior to attending a subsequent event, where alcohol consumption often continues [8, 9]. Pre-drinking is also referred to as *prepartying* [8], *pregaming* [9], and *pre-loading* [10]. Pre-drinking has been found to constitute more than 40 % of alcohol consumption on drinking occasions [11], and an Australian multi-site study conducted in night entertainment areas found 65 % of people reported pre-drinking prior to ‘going out’ for that evening [12]. Pre-drinking has been shown to be largely socially-motivated, with pre-drinkers citing “catching up” with friends and meeting new people as precipitating factors contributing to the popularity of these sessions [13–15]. LaBrie et al. [15] found that *interpersonal enhancement* (i.e., pre-drinking for socialisation or enjoyment) was the strongest predictor of pre-drinking frequency and alcohol consumption, and demonstrated that pre-drinking motives differ from general alcohol consumption motives. Alcohol price has also been shown to be related to pre-drinking. Not only have students cited cost as influencing their pre-drinking [11, 16], but Miller and Droste [17] have shown that students change their hypothetical drinking decisions based on increases in the cost per drink. A recent study shows a relationship between strongly endorsing a cost motive for pre-drinking, and higher reported typical pre-drinking consumption [18].

In a series of recent studies, pre-drinking has been implicated as specifically contributing to alcohol-related harm. An event-level analysis by Barry et al. [19] found pre-drinking status significantly predicted blood-alcohol concentration, as measured by a breathalyser device. Merrill et al., [20] used event-level associations to reveal that pre-drinking on any given day was a significant predictor of alcohol related harm in university students, beyond both the total alcohol consumed on that day, and typical drinks consumed per day. In a sample of undergraduates, Caudwell and Hagger [18] found higher scores on pre-drinking cost motive items predicted higher incidence of alcohol-related harm in the previous twelve months. Pre-drinking appears to present an elevated risk to young adults, who demonstrate a lack of awareness of safe alcohol consumption limits [21], and, in laboratory settings, are unable to accurately pour a standard drink^1^ [22, 23]. To date, no interventions specifically aimed at reducing pre-drinking alcohol consumption have been developed. This protocol outlines a theory-based intervention that will attempt to reduce alcohol consumption during pre-drinking sessions, and the experience of alcohol-related harm.

### Theory-based interventions for excessive alcohol consumption

One approach to reducing excessive alcohol consumption and alcohol-related harm among undergraduates is to develop behavioral interventions based on social psychological and motivational theories of health behavior. The use of such theories in informing interventions is important in targeting the influential determinants of health behavior, facilitating an understanding of “what works, and for whom”, and allows for testing of the component theories in accounting for behavior change [24]. A range of health behavioral interventions targeting excessive alcohol consumption have been developed in university student populations, incorporating brief screening and feedback [25], motivational [26–28], peer or normative feedback [29–31], planning [32], and volitional approaches [33–35]. Though the efficacy of online interventions appears to bring about small changes in alcohol consumption behaviour [d + = 0.14; [36]], many interventions are not theory-based, and there is evidence that theory-based interventions that closely develop intervention content to target specific psychological variables (commonly identified as correlates or predictors of alcohol consumption) are efficacious, with medium-sized effects [37, 38]. Furthermore, evidence supports the use of online delivery of alcohol interventions in student populations as they appear preferable to face-to-face methods (e.g., contact with a health professional) and may be especially useful for at-risk populations [39, 40]. Therefore, the development of a theory-based online intervention to reduce pre-drinking alcohol consumption may be a useful endeavour.

### The theory of planned behavior

The *theory of planned behaviour* [41] has been extensively applied to predict a range of health behaviours [42–44]. The theory considers behavioural *intention* the focal point of behavioural engagement, where *intention* is formed by belief-based constructs of *attitude*, *subjective norm*, and *perceived behavioral control* [41]. *Attitude* comprises belief-based evaluations of the behavior of interest; *subjective norm* consists of perceived social influence regarding behavioural engagement, and; *perceived behavioral control* constitutes the individual’s ability to perform the behavior. The theory has been widely used across a range of health behavioural contexts, with a recent meta-analysis supporting the tenets of the theory-based model in predicting intention and behavior [44]. More recently, a meta-analysis of the theory applied to alcohol consumption behaviour has found attitudes strongly related to alcohol consumption intentions (r_+_ = .62), and intentions moderately related to behaviour (r_+_ = .54) with authors concluding that both attitudes and intentions towards alcohol consumption are worthwhile targets for alcohol consumption behaviour change [45]. Generally, changes in behavioral intention appear to produce small-to-moderate changes in behaviour [46], with theory-based health behavioral interventions informed by the theory of planned behavior demonstrating particular efficacy [d + = 0.36; [36]], supporting our advocacy of adopting a theoretical approach.

A prominent criticism of the theory is the *intention-behavior gap*: the relative weakness in the link between intention and behaviour [47–50]. This is an important issue for interventions where intention may be the focus, yet it is a weak or modest predictor of behavioural engagement. For example, McEachan, Conner [44] shows the intention-behaviour relationship is weaker for health risk behaviours, such as abstaining from alcohol consumption, compared to health enhancing behaviors such as diet and exercise. A recent meta-analysis investigating the relationships between the theory of planned behaviour constructs applied to alcohol consumption concluded that interventions targeting attitudes and subjective norm may be worthwhile [45]. However, there is little utility in attempting to change intention through its antecedent constructs, where a substantial intention-behaviour gap is unlikely to facilitate meaningful behaviour change. This point and the utility of the theory of planned behaviour in health behavioural research is one of current debate (see [50]), with Schwarzer [51] suggesting that post-intentional (i.e., volitional) constructs that are known to influence behaviour are of importance in interventions based on the theory of planned behaviour. *Implementation intentions* [52] present an approach to “closing” the intention-behavior gap by linking important contextual cues to enacting the intended behaviour in the volitional stage, increasing the likelihood that the behavior is carried out in accordance with one’s intentions.

### Implementation intentions and volition

According to Gollwitzer [53], individuals who intend to reach an intended goal often fail to do so due to limitations in their ability to self-regulate behaviour. These limitations may constitute reasons such as failing to get started (e.g., forgetting or failing to act at the opportunity to do so) and getting derailed (e.g., due to attentional or competing factors; Gollwitzer & Sheeran, [54]). For example, a pre-drinking goal intention may be “I intend to reduce my alcohol consumption drinking during pre-drinking sessions”. However, an individual with this intention may not recognise the chance to enact that intention or fail to do so at the critical moment (e.g., where an environment is conducive to excessive alcohol consumption). *Implementation intentions* increase the likelihood that people will attain their intended goals by specifying contextual details of how these goals will be implemented, as well as when, and where [55]. An implementation intention for pre-drinking may therefore be “when I have finished an alcoholic drink at a pre-drinking session, I will then drink a glass of water or soft drink to help reduce my alcohol consumption”. This allows individuals to switch from making conscious, effortful deliberations about enacting behaviour, to responding automatically to critical cues [52], mitigating the effects of self-regulatory limitations on carrying out intended behaviours. A meta-analysis by Gollwitzer and Sheeran [54] shows that there is a considerable effect (*d*_*+*_ = .65) of implementation intentions in facilitating goal attainment over that of simply forming goal intentions. Importantly, implementation intention approaches have been shown to be effective in reducing alcohol consumption in young people including university students [35, 38, 56].

Key features of an implementation intention approach include detailing *how* the intended behaviour will be enacted. In previous studies using this approach, participants either formed their own implementation intentions [38] or chose from a menu of responses to refusing a drink with the option of developing their own plan [35]. These studies and a recent review by Hagger and Luszczynska [57] suggest that implementation intentions may be more successful if they include additional planning components that address certain contingencies in an *if-then* format, such as “*if* I am offered an alcoholic drink, *then* I will politely refuse by saying, ‘No thanks, I have to drive” [35]. In the context of pre-drinking, there are likely many contextual scenarios where individuals may be at risk of consuming excessive amount of alcohol (e.g., drinking games, coercion or pressure) [9, 58]. Therefore, the formation of multiple implementation intentions to address these scenarios may be especially effective in reducing pre-drinking alcohol consumption. However, compelling individuals to intend to perform certain behaviours and assisting them in doing so may not be as effective if individuals lack the necessary motivational resources to facilitate the formation of these intentions and subsequent behavior.

### Self-determination theory

Another theoretical framework that has seen wide application in many health-related fields is *self-determination theory* [59–62]. Self-determination theory places the *quality* of an individuals’ motivation as influential in behavioural engagement and persistence. Individuals who exhibit *controlled motivation* to engage in a behaviour tend to do so because of certain external contingencies - monetary incentive or reward, or for self-esteem rationales such as avoiding guilt or blame, or embarrassment [59]. Individuals who exhibit *autonomous motivation* to engage in a behaviour tend to do so because it serves personally-relevant goals or the act is itself intrinsically rewarding [59]. The more autonomously motivated an individual is towards engaging in behaviour, the more likely they will be to perform and persist in performing that behaviour [63, 64]. Recent evidence indicates attitudes and intentions towards engaging in health behaviour are more strongly linked to autonomous motivation rather than controlled motivation [65, 66].

Health behavioural interventions based on self-determination focus on the facilitation of autonomous motivation [60, 67]. This is often achieved by providing *autonomy support –* a supportive context and rationale for the individuals’ internalising of behavioural regulation [63]. The provision of autonomy support and facilitation of autonomous motivation have demonstrated validity in engendering positive behavioural change in a wide context of health behavioural settings [62]. Within the context of alcohol consumption, studies involving self-determination theory have found relationships between autonomous forms of motivation and reductions in self-reported alcohol consumption [68], as well as intentions to keep alcohol consumption within limits, and reductions in alcohol units consumed [69]. Pavey and Sparks found that autonomy in relation to perceptions of health risk information and autonomous motivation to engage in health protective behaviours were related to participation in those behaviours [70–72].

Conversely, studies on peer influences in college drinkers have shown individuals who exhibit controlled motivation to drink excessively do so because they tend to appraise situations from a controlled orientation, related to their sense of self-esteem [73]. Therefore, an intervention that provides an autonomy-supportive context for reduced alcohol consumption may prove effective for pre-drinkers who consume alcohol excessively or in contexts where motivation to reduce excessive alcohol consumption may be lacking. Given research demonstrating the importance of autonomy in enhancing receptiveness to health risk information, and indicating intrinsic goals are more likely to be pursued than those where individuals feel compelled to pursue goals [64, 70, 72], individuals may be more autonomously motivated to reduce their pre-drinking alcohol consumption if they generate their own autonomous reasons for pursuing such a goal.

### Evidence for combining approaches

A meta-analysis of internet-based health behavioral interventions has found those incorporating more behavior change techniques tended to have larger effects, potentially due to these techniques targeting different components of the behaviour change process [36]. According to the model of action phases proposed by Heckhausen and Gollwitzer [74], a “Rubicon” exists between a deliberative, or *predecisional* phase, and a volitional, or *preactional* phase. The predecisional phase incorporates the feasibility and desirability of a behavioral outcome; the motivational tendency towards enacting that behavior which leads to the formation of a *goal intention* [75]. The preactional phase therefore incorporates how best to meet the behavioral goal – the stage at which individuals may fall short of meeting that goal due to limitations in their ability to self-regulate behavior [75]. It follows, therefore, that interventions targeting both motivation and volitional phases of action may be more effective in evoking behaviour change.

Studies have also shown that intentions are more likely to be carried out if they are formed consistent with autonomous reasons for engaging in the target behavior [76] and when the behavior is consistent with their psychological needs [77]. Evidence shows support for a synergistic relationship between autonomous motivation and the formation of implementation intentions in facilitating goal-directed behaviour. For example, a study on goal *self-concordance* (i.e., the extent to which a goal-directed behaviour is self-determined), found self-concordance significantly predicted progress on a range of participant goals, and that the relationship between goal self-concordance and progress was dependent on whether or not participants formed implementation intentions [78]. Koestner et al. [79] demonstrated that participants who formed autonomy-supportive implementation intentions achieved greater goal progress than those in a neutral condition (*d* = .67). The authors attribute this to the internalisation of goals in a self-concordant manner that reflects heightened personal interest and meaning. In terms of interventions based on this premise, targeting the motivational and volitional phases in tandem show increased efficacy in reducing alcohol consumption [33], promoting exercise behavior [80], reducing saturated fat intake [81], and improving fitness [82] over either approach in isolation.

### The present study

The purpose of the present study is to test an online, theory-based intervention to reduce pre-drinking alcohol consumption among undergraduate students who pre-drink. The intervention will test the effects of two theory-based techniques targeting the predecisional and implemental phases of the model of action phases through: (1) facilitating autonomous motivation to reduce pre-drinking alcohol consumption, and (2) prompting the individual to form context-specific implementation intentions to help bridge the goal intention-behavior gap. Combining these techniques should see greater reductions in pre-drinking alcohol consumption and alcohol-related harm than either approach in isolation. The current research makes an original contribution to knowledge by adopting a factorial design, which permits us to examine the independent and interactive effects of two intervention components related to different processes in the model of action phases. The research builds on previous approaches to promoting autonomous motivation [79] and based on current ‘best practice’ recommendations for using implementation intentions [57]. It also follows on from research that suggests that incorporating both motivational and implemental phases is optimally effective in changing health behaviour by targeting multiple processes [38, 80, 81].

## Methods

### Design

**Fig. 1:**
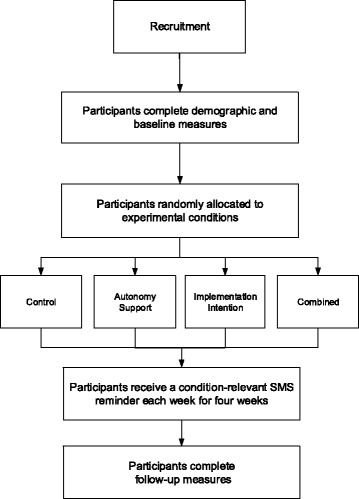
Conceptual diagram showing the intervention components and their influence on the two stages of action [74]. ^1^National Health and Medical Research Council guidelines for reducing risk related to alcohol consumption

The study will adopt a 2 (autonomy support: present vs. absent) x 2 (implementation intention: present vs. absent) design (see Fig. 1). Given evidence for the use of periodic prompts in supporting online interventions [83, 84] and the increased effectiveness of presenting reminders in implementation intention interventions [85] participants will be sent the components of their respective intervention via email following its conclusion. At follow-up, four weeks later, participants will be invited to complete the same theory-based measures as at baseline to assess the influence of the intervention in terms of changes in theoretical constructs and behavior.

**Fig. 2:**
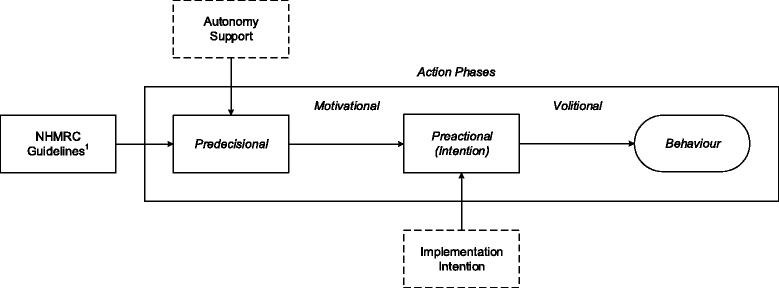
Flow diagram detailing participant progress through the study

### Intervention components

Participants will be randomly allocated to one of four conditions: a control condition, an autonomy support condition, an implementation intention condition, and a combined autonomy support and implementation intention condition. Each condition will include the first two guidelines of the Australian National Health and Medical Research Council safe drinking guidelines [1]. These guidelines are recommendations for keeping alcohol consumption within limits to reduce the risk of alcohol-related harm over the lifetime, and are included in the Appendix. (Fig. 2) shows the intervention components alongside the intended action phase targets. 

#### Autonomy support condition

Participants will be asked to generate statements that reflect a series of interpersonal conditions of autonomy support, as outlined in Su and Reeve [86]. These are closely based on verified approaches used throughout self-determination theory-based interventions to facilitate autonomous motivation to engage in the target behavior [86–89]. The five conditions outlined in Su and Reeve [86] include: providing meaningful rationales (i.e., why self-regulated engagement in reducing pre-drinking alcohol consumption may be beneficial)*,* acknowledging negative feelings (i.e., feelings associated with reducing pre-drinking alcohol consumption); use of non-controlling language *(e.g., may* or *could* rather than *must* or *should*); offering choices (i.e., promoting choice-making and encouragement)*,* and nurturing inner motivational resources (i.e., making the satisfaction of needs for autonomy, competence, and relatedness salient in the communication)*.* Table 1 includes example prompts and statements to be used in the intervention.

#### Implementation intention condition

Participants will be informed of how forming specific *if-then* plans to reduce their alcohol intake during pre-drinking sessions can assist them in doing so. Given that personally-relevant goals have been found more effective in leading to behavioral engagement [90], and the importance of self-relevant cues in leading to action, as outlined in Heckhausen and Gollwitzer [74], participants will be asked to detail a series of situations in which they might be at risk of excessive pre-drinking alcohol consumption. Participants will then be provided with examples of implementation intentions before being asked to generate their own that correspond to their identified situations, using two (i.e., *if*…, *then*…) open-response text boxes [57].

### Combined condition

Heckhausen and Gollwitzer’s [74] action-phase model places the predecisional (i.e., motivational) as preceding a behavioural decision, from which an individual passes through to the preactional (i.e., volitional) phase. Accordingly, participants in the combined condition will first receive the autonomy support component, followed by the implementation intention component. A conceptual map of the intervention components relative to the components in the action-phase model is included in Fig. 2.

### Measures

#### Theory of planned behaviour

Attitude, subjective norm, and perceived behavioural control items will be used based on previous research [38, 91]. *Attitude* will be measured with a common item stem (i.e., “*reducing alcohol consumption during pre-drinking sessions would be*…”) followed by a series of five bipolar adjectives (e.g., *bad-good, beneficial-harmful*), with participants asked to score each adjective accordingly on a six-point scale*. Subjective norm* will be measured with three statements referring to perceived pressure from others to engage in pre-drinking (e.g., “*people who are important to me would want me to reduce my alcohol consumption during pre-drinking sessions”)* with participants asked to respond to each on six-point Likert-type scales ranging from 1 (*strongly disagree*) to 6 (*strongly agree*). *Perceived behavioural control* will be measured with three statements regarding control (e.g., *If I wanted to, I could reduce my alcohol consumption during pre-drinking sessions*), with participants asked to respond to each on six-point Likert-type scales ranging from 1 (*strongly disagree*) to 6 (*strongly agree*). *Intention* to reduce pre-drinking alcohol consumption will be measured with three items (e.g., *I will reduce my alcohol consumption during pre-drinking sessions*) with six-point Likert-type scales ranging from 1 (*strongly disagree*) to 6 (*strongly agree*).

#### Planning

Nine items from the planning subscale of the Self-Regulation Questionnaire [92] will be used to measure participants’ planning ability. Participants will respond to these items (e.g., “*I have trouble making plans to help me reach my goals*”) on six-point Likert-type scales ranging from 1 (strongly disagree) to 6 (strongly agree).

#### Autonomous motivation and goal self-concordance

Sheldon and Kasser [77] have developed a measure of goal self-determination, whereby participants rate how much they pursue goals for specific controlled, non-self-determined reasons (e.g., “…*because somebody wants me to, or because I’ll get something from somebody if I do*”, “*I probably wouldn’t do this if I didn’t get some kind of reward, praise, or approval for it*”), or autonomous, or self-determined reasons (e.g., *because I really believe that it is an important goal to have – I endorse it freely and value it wholeheartedly*). Participants will respond on nine-point Likert-type scales ranging from 1 (*not at all for this reason*) to 9 (*completely because of this reason*). Controlled scores are subtracted from autonomous scores to derive a relative score for goal self-concordance [76].

#### Goal progress

Participants will be asked to rate the extent of their progress, if any, in reducing their alcohol consumption during pre-drinking sessions, on a nine-point Likert-type scale ranging from 1 (*none at all*) to 9 (*total progress*), as used in previous research [78].

#### Pre-drinking alcohol consumption

Participants will report their pre-drinking alcohol consumption in terms of Australian standard drink equivalents consumed during pre-drinking sessions each week, over the previous four weeks, with the aid of a pictorial guide [1], at both baseline and follow-up. The pictorial guide comprises examples of typically served or available portion sizes of alcoholic beverages (e.g., a carton of beer, a bottle of wine or spirits) to aid in participant estimation of pre-drinking alcohol consumption (i.e., pre-purchased quantities such as bottles of spirits or cartons of beer). This approach has been used in previous research [91].

#### Alcohol-related harm

The Brief Young Adult Alcohol Consequences Scale (B-YAACQ) [93] is a validated measure of the experience of alcohol-related harm that is well-suited to use in college populations for the purpose of evaluating change in alcohol consequences. The measure comprises a series of 24 participant-endorsed yes/no statements related to alcohol-related harm (e.g., “*I have felt very sick to my stomach or thrown up after drinking”*). Scores are derived from summing all *yes* responses to create a unidimensional index of alcohol-related harm [93]. Participants will complete the B-YAACQ at baseline and follow-up, to ascertain the effects of the intervention in reducing alcohol-related harm attributable to reductions in pre-drinking alcohol consumption. The time-frame of the B-YAACQ will be modified to refer to harm from alcohol consumption in the previous four-week period, to give a fine-grained view of the effects of the intervention on alcohol-related harm (see [94]).

### Hypotheses

It is hypothesized that participants receiving both autonomy support and implementation intention components will exhibit greater reductions in pre-drinking alcohol consumption and alcohol-related harm at follow-up, relative to participants receiving either intervention component in isolation, in accordance with evidence supporting the combination of these approaches in potentially targeting two important components of the action-phase model [64, 75, 76].

### Participants

Eligible participants will be current undergraduate students who regularly consume alcohol (i.e., are current ‘drinkers’), and have engaged in pre-drinking behaviour within the previous six months. Based on medium effects for implementation intentions on reductions in alcohol consumption reported in Hagger, Lonsdale [33] and the meta-analysis of self-determination theory applied to health contexts reported in Ng et al. [62], we conducted a statistical power analysis using G*Power to ascertain an adequate sample size for the intervention. Specifically, the power analysis was for an Analysis of Covariance (ANCOVA) on the two key dependent variables, alcohol consumption and summed B-YAACQ scores, with the intervention groups as the independent variables powered to detect a medium effect size (Cohen’s *f* = .25) with power set at .80 and alpha set at .025, and baseline scores on the dependent variable as a covariate. The analysis yielded 196 participants (i.e., 49 per group) for each analysis.

### Analyses

#### Randomisation check

A 2 (autonomy support: present or absent) x 2 (implementation intention: present or absent) MANOVA will be conducted, with baseline demographic, behavioural, and psychological measures as dependent variables, and the intervention components as the independent variables, to test for between-group differences across the intervention conditions at baseline.

#### Manipulation checks

As the effect of implementation intentions might be diminished by participants failing to comply with instructions consistent with the approach, we will content analyse participants’ implementation intention scripts (typed in response to the implementation intention manipulation) to ascertain the extent to which participants complied with the intervention instructions [33]. Independent raters familiar with implementation intentions will rate the quality of scripts based on the presence or absence of key planning components: (1) used the if-then format, (2) specified a relevant, realistic, and appropriate cue, (3) linked the cue to the desired response. A one-way independent groups ANOVA will be conducted to test the effect of autonomy support on goal self-concordance as a manipulation check.

#### Effects of the intervention on pre-drinking alcohol consumption and alcohol-related harm

Two ANCOVAs (autonomy support: present or absent) x 2 (implementation intention: present or absent) will be conducted to ascertain the effect of the intervention on follow-up self-reported pre-drinking alcohol consumption, and summed B-YAACQ scores, at follow-up, controlling for baseline pre-drinking alcohol consumption.

#### Effects of the intervention on psychological variables

A 2 (autonomy support: present vs. absent) x 2 (implementation intention: present vs. absent) MANCOVA will be conducted, with autonomous motivation, constructs from the theory of planned behaviour (attitudes, subjective norm, perceived behavioural control, and intention) and goal progress as dependent variables, and pre-intervention pre-drinking alcohol consumption as a covariate.

### Ethics

The study protocol was approved by Curtin University Research Ethics Committee (HR185/2014/AR1). Participants will provide informed consent to participate in the intervention.

## Discussion

Pre-drinking is associated with significant risks attributable to excessive alcohol consumption [19, 20, 95]. No theory-based interventions to reduce pre-drinking alcohol consumption have yet to be developed. The present protocol has outlined a theory-based intervention that will attempt to reduce pre-drinking alcohol consumption and alcohol-related harm, by targeting the volitional and motivational phases of action, according to the action-phase model outlined by Heckhausen and Gollwitzer [74]. There is evidence that the provision of autonomy support is associated with greater autonomous motivation to engage in behaviour, and that autonomous motivations for reducing alcohol consumption are associated with reductions in alcohol consumption [33, 69]. Although, exhibiting motivation is a necessary but not sufficient condition for behavioural enactment [49, 96]. Forming implementation intentions has been shown to strengthen the link between intention and behaviour, by providing a link between a contextual cue and an intended response [35, 54]. Combining these approaches is based on the premise that promoting goal self-concordance is important in successful goal attainment [78], and is integral to the efficacy of implementation intention approaches [57, 79]. Therefore, an approach that combines the volitional and motivational action phases, providing individuals with autonomy supportive context for behaviour change and the regulatory skills with which to translate this motivational impetus into behaviour, may be more effective in eliciting successful behaviour change. We therefore expect that while participants in the autonomy support and implementation intention conditions will report lower pre-drinking alcohol consumption at follow-up, the combination of these approaches will see the greatest reduction in pre-drinking alcohol consumption. This is because individuals may be autonomously motivated and intend to reduce their pre-drinking alcohol consumption, let may lack the regulatory capacity required to translate this intention into action (i.e., inclined abstainers) [48]. Similarly, the formation of if-then plans to reduce pre-drinking alcohol consumption may not lead to action if the underlying rationale for these plans is not autonomous [79]. Providing autonomy support to facilitate autonomous motivation will form a sound basis for the development of if-then plans, leading to the translation of that motivational basis into successful action and greater reductions pre-drinking alcohol consumption.

There are some limitations in the design of the intervention that should be noted. As the intervention will be delivered online, there may be potential problems with attrition between baseline and follow-up [33]. This has the potential to reduce the statistical power of the intervention to detect an effect, and limit testing intervention effects on the relevant theoretical constructs. To mitigate this, recruitment will attempt to account for the attrition rate observed in recent online interventions [33, 97]. It is important to note that the primary focus of the intervention is to ascertain the overall efficacy of the intervention conditions in terms of reductions in the primary outcome variables, pre-drinking alcohol consumption and alcohol-related harm, rather than the mediating effects of theoretical constructs which are important issues but secondary to overall effects. Secondly, reviews of alcohol interventions often cite the lack of continued follow-up as detrimental to establishing the efficacy of these interventions over time [40, 98]. However, the efficacy of this intervention can be considered a basis for further research that ascertains the extent of intervention efficacy over time. Finally, there are many issues with the validity and accuracy of self-reported alcohol consumption [99]. However, by using pictorial aids detailing standard drink equivalents for commonly-consumed alcoholic beverage containers [1], we attempt to mitigate errors in measurement. Further, by measuring goal attainment, the effect of the intervention on fulfilling participant goals to reduce pre-drinking alcohol consumption and alcohol-related harm can also be assessed.

## Appendix

### Intervention components

#### Control condition

Participants are presented with two guidelines from the NHMRC [1] to inform them of the relationship between alcohol consumption and the risk of alcohol-related harm over a lifetime, and reducing the risk of injury on a single occasion of drinking.

GUIDELINE 1

Reducing the risk of alcohol-related harm over a lifetime.

The lifetime risk of harm from drinking alcohol increases with the amount consumed.

For healthy men and women, drinking no more than two standard drinks on any day reduces the lifetime risk of harm from alcohol-related disease or injury.

GUIDELINE 2

Reducing the risk of injury on a single occasion of drinking.

On a single occasion of drinking, the risk of alcohol-related injury increases with the amount consumed.

For healthy men and women, drinking no more than four standard drinks on a single occasion reduces the risk of alcohol-related injury arising from that occasion.

### Autonomy support condition

Participants are presented with a script comprising a series of statements using autonomy-supportive language, and given prompts to write about reasons pursuing the goal of reducing their pre-drinking alcohol consumption may be worthwhile:

“Over the next few weeks, we’d like you to consider reducing your pre-drinking alcohol consumption. Remember, when we talk about pre-drinking, we mean: drinking alcohol at home, or someone else’s house, prior to attending an event (where drinking alcohol may continue).

Pre-drinking can be harmful, so there are many good reasons why people might set themselves the goal of reducing their pre-drinking alcohol consumption. While we understand that this goal may not be overly enjoyable or interesting, if you identify reasons why reducing your pre-drinking alcohol consumption is important to you, you may feel you are more able to meet this goal. You are free to choose exactly how you will reduce your pre-drinking alcohol consumption – developing your own strategy that uses your set of skills and resources often leads to success.

The following prompts are to help you think of ways you can reduce your pre-drinking alcohol consumption, avoid negative outcomes associated with alcohol consumption, and gain the benefits of reducing pre-drinking alcohol.

You will be taken through these ways step-by-step; as you read, you will be provided with reasons for completing these prompts and how they might help you.

Remember, whether or not you engage in these exercises is entirely up to you - it’s your choice.

Example:

Identifying some of the negative consequences of pre-drinking can be a good first step in forming your plan to reduce your pre-drinking alcohol consumption. Reducing your alcohol consumption during pre-drinking sessions will help you to avoid some of these negative consequences you may experience when pre-drinking excessively.”

### Implementation Intention Condition

Participants are told they are more likely to reach their intended goal of reduced pre-drinking alcohol consumption if they think of “if-then plans” that specify when and where these plans will be enacted:

“You are more likely to carry out your intention to reduce the amount of alcohol you consume during pre-drinking sessions if you make a decision about the time and place you will do so, and how you plan to do it.

Decide now when and where you will need to limit the amount of alcohol you consume during pre-drinking sessions, and how you will do it. We want you to plan to reduce the pre-drinking alcohol you consume over the next month, paying particular attention to the specific situations in which you will need to implement these plans.

For example, you might find it useful to say to yourself: *“*When I finish an alcoholic beverage during a pre-drinking session, I will then drink a glass of water to help limit my alcohol consumption*.*”

Alternatively, you might find it useful to say to yourself: “When I am offered a drink during a pre-drinking session, I will say, “No thanks, I have to get up early tomorrow.”

Example:

Please choose from the options below, or write your plans in the text box available, following the format shown in the previous example (i.e., if… then…). Remember, it is important to remember the specific situation in which you need to implement your plan.”

## Footnotes

1 In Australia, a standard drink is defined as an alcoholic beverage containing 10 g of ethanol (alcohol).
